# Phytochemical Study of Aerial Parts of *Leea asiatica*

**DOI:** 10.3390/molecules24091733

**Published:** 2019-05-04

**Authors:** Hyun Woo Kil, Taewoong Rho, Kee Dong Yoon

**Affiliations:** College of Pharmacy and Integrated Research Institute of Pharmaceutical Sciences, The Catholic University of Korea, Bucheon 14662, Korea; kilhyunwoo@catholic.ac.kr (H.W.K.); karlwho@naver.com (T.R.)

**Keywords:** *Leea asiatica*, leeaceae, phytochemistry, triterpenoids, phenolic glyscosides

## Abstract

*Leea asiatica* (*L.*) Ridsdale (Leeaceae) is found in tropical and subtropical countries and has historically been used as a traditional medicine in local healthcare systems. Although *L. asiatica* extracts have been found to possess anthelmintic and antioxidant-related nephroprotective and hepatoprotective effects, little attention has been paid toward the investigation of phytochemical constituents of this plant. In the current study, phytochemical analysis of isolates from *L. asiatica* led to the identification of 24 compounds, including a novel phenolic glucoside, seven triterpenoids, eight flavonoids, two phenolic glycosides, four diglycosidic compounds, and two miscellaneous compounds. The phytochemical structures of the isolates from *L. asiatica* were elucidated using spectroscopic analyses including 1D- and 2D-NMR and ESI-Q-TOF-MS. The presence of triterpenoids and flavonoids supports the evidence for anthelmintic and antioxidative effects of *L. asiatica.*

## 1. Introduction

**Figure 1:**
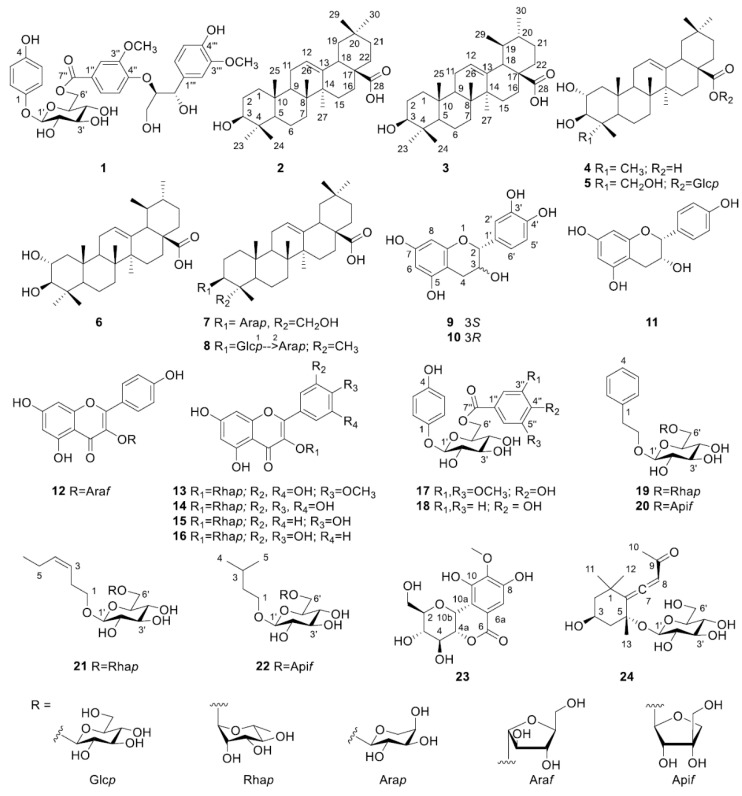
The chemical structures of compounds **1**–**24** from the aerial parts of *Leea asiatica.*

Plant species of the genus Leeaceae (*Leea)* are widely distributed among tropical and subtropical regions, including Eastern Africa, Australia, China, India, Indonesia, Laos, Malaysia, Myanmar, and Thailand [1,2]. Around 70 different species of the genus *Leea* (*L.*) are found in these regions, with some being used in local traditional healthcare systems to treat bone fractures, liver disorders, physical wounds, skin diseases, and worm infections [1]. Various biological studies have reported the antibacterial, antioxidative, anthelmintic, cytotoxic, hepatoprotective, and nephroprotective effects of the *Leea* species. Several phytochemicals, including coumarin, essential oils, flavonoids, hydrocarbons, and triterpenoids have been determined from the extracts of diverse *Leea* species, such as *L. thorelii* [1,3], *L. indica* [4,5,6,7,8,9], *L. macrophylla* [10,11], and *L. guineense* [12,13]. Although several *Leea* species have been evaluated from both a biological and phytochemical perspective, relatively few studies have included *L. asiatica* (*L.*) Ridsdale in their analyses. *Leea asiatica* has also been used as a folk medicine, especially in India, to treat worm infection, bone fracture, liver disorder, and oxidative stress-related diseases. Up to date, three published reports have described the biological effects of *L. asiatica* extracts, including anthelmintic and antioxidant-related nephroprotective and hepatoprotective activities [14,15,16], and no phytochemical study on this species has been attempted yet even though *L. asiatica* has been used as a traditional medicine, as have other *Leea* species. The identification of the phytochemicals of herbal medicine is very important because it can be used as a primary data for the prediction of biological effects, safety information, and clarification of medicinal use. From this point of view, the current study was designed to identify chemical constituents of *L. asiatica* and to provide their spectroscopic information. We describe here a novel compound (**1**) along with seven triterpenoids (**2**–**8**), eight flavonoids (**9**–**16**), two phenolic gluocosides (**17**–**18**), four diglycosidic compounds (**19**–**22**), and two miscellaneous compounds (**23**–**24**) (Figure 1).

## 2. Results and Discussion

### 2.1. Elucidation of Chemical Structures of Compounds ***1***–***24***

**Figure 2:**
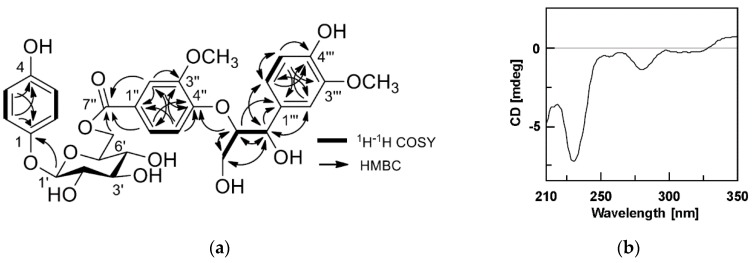
(**a**) ^1^H-^1^H COSY and HMBC correlation of compound **1**; (**b**) circular dichroism (CD) spectrum of compound **1**.

The molecular formula of compound **1** was identified as C_30_H_34_O_14,_ according to its ESI-Q-TOF-MS spectrum, which showed the quasi-molecular ion peak at *m/z* 617.1884 [M − H]^−^ −(calcd. for C_30_H_33_O_14_, 617.1870). The ^1^H-NMR spectrum of compound **1** showed resonances characteristic for a 1,4-disubstituted benzene ring [δ_H_ 6.91 (2H, d, *J* = 9.0 Hz, H-2, 6), 6.56 (2H, d, *J* = 9.0 Hz, H-3, 5)], an anomeric proton of a sugar group at δ_H_ 4.73 (1H, d, *J* = 7.4 Hz, H-1′), two sets of 1,3,4-trisubstitued benzene moieties [δ_H_ 7.57 (1H, dd, *J* = 8.5, 2.0 Hz, H-6′′), 7.51 (1H, d, *J* = 2.0 Hz, H-2′′), and 7.06 (1H, d, *J* = 8.5 Hz, H-5′′); δ_H_ 7.07 (1H, o, H-2′′′), 6.87 (1H, dd, *J* = 8.1, 2.2 Hz, H-6′′′), and 6.71 (1H, d, *J* = 8.1 Hz, H-5′′′)], two oxygenated methine signals at δ_H_ 4.84 (1H, o, H-7′′′) and 4.64 (1H, m, H-8′′′), a hydroxylated methylene resonance at δ_H_ 3.86 (2H, brd, *J* = 4.9 Hz, H-9′′′), and two methoxy signals [δ_H_ 3.79 (3H, s, 3′′-OCH_3_) and 3.80 (3H, s, 3′′′-OCH_3_)]. Exhaustive 1D- and 2D-NMR (^1^H-^1^H COSY, HSQC, and HMBC) interpretations suggested that compound **1** contained a guaiacylglycerol 8-yl, a 3-methoxy-4-hydroxyphenoxy, a 4-hydroxy-benzene, and a β-glucopyranosyl moiety, as shown in Figure 1. The sugar unit was identified as D-glucose by acid hydrolysis. The connectivity of each functional group was established using the HMBC spectrum. Crossed peaks were observed at δ_H_ 4.73 (H-1′)/ δ_C_ 152.3 (C-1), δ_H_ 4.68, 4.37 (H-6′a and H-6′b)/ δ_C_ 167.7 (C-7′′), and δ_H_ 4.64 (H-8′′′)/ δ_C_ 154.2 (C-4′′) (Figure 2a). The 7′′′,8′′′-*erythro* configuration of compound **1** was deduced from the *Δ*δ_C8′′′- C7′′′_ value (11.0 ppm), and a negative Cotton effect at 229 nm in its circular dichroism (CD) spectrum indicated the 8*R* configuration, according to the findings of previous reports (Figure 2b) [17,18]. Based on the spectroscopic evidence (Supplementary Information SI 7–SI 8), and searching the confirmed structure of compound **1** through Scifinder^®^ and Reaxys^®^ databases, compound **1** was identified as a new compound with a chemical structure of 4-hydroxyphenol-β-D-{6-*O*-[4-*O*-(7*S*,8*R*-guaiacylglycerol-8-yl)-3-methoxybenzoyl]}-β-D-glucopyranoside. The molecular structure of 1 was very similar to that of (−)-4-hydroxy-3-methoxyphenol β-D-{6-*O*-[4-*O*-(7*S*,8*R*)-(4-hydroxy-3-methoxyphenylglycerol-8-yl)-3-methoxybenzoyl]}-glucopyranoside, which was determined in a previous phytochemical study [16]. The comparison of ^1^H- and ^13^C-NMR data of both compounds was described in Supplementary Information SI 8.

The following known compounds were also identified: Oleanolic acid (**2**) [19], ursolic acid (**3**) [19], maslinic acid (**4**) [20,21], chebuloside ii (**5**) [22,23], corosolic acid (**6**) [21], hederagenin-3-*O*-arabinopyranoside (**7**) [24], oleanolic acid 3-*O*-glucopyranosyl-(1→2)-arabinopyranoside (**8**) [25], (+)-catechin (**9**) [26], (−)-epicatechin (**10**) [26], (−)-epiafzelechin (**11**) [27], juglanin (**12**) [28], mearnsetin 3-*O*-rhamnopyranoside (**13**) [29], myricitrin (**14**) [30], afzelin (**15**) [30], quercitrin (**16**) [30], 4-hydroxyphenol-[6-*O*-(4′′-hydroxy-3′′,5′′-dimethoxy-benzoate)]-β-d-glucopyranoside (**17**) [31], breynioside A (**18**) [32], phenylethyl-rutinoside (**19**) [33], icariside D1 (**20**) [34], hexenyl-rutinoside (**21**) [35], everlastoside C (**22**) [36], bergenin (**23**) [37], and citroside A (**24**) [38]. To the best of our knowledge, compounds 2 [9] (*L. macrophylla*), 3 (*L. indica*) [5], 16 [3,5,9] (*L. indica* and *L. thorelii*), **9**, **10**, **14**, **16**, and **24** (*L. inidca* and *L. thorelii*) [3] have been previously isolated from the several other *Leea* species, whereas compounds **4**–**8**, **11**–**13**, **15**, and **17**–**22** were here isolated from the *Leea* species, especially from *L. asiatica*, for the first time. However, the phytochemical profile of the plant is mainly affected by the genetic and environmental factors (e.g., soil, climate conditions, and seasonality) during plant growth, extraction, and isolation method. This phytochemical study does not represent all chemical profiles of *L. asiatica*. Therefore, further phytochemical study on *L. asiatica* from different regions should be performed to achieve a full understanding of the chemical composition of *L. asiatica*.

As mentioned in the Introduction, *L. asiatica* possessed anthelmintic [14] and antioxidative activity-related nephroprotective and hepatoprotective effects [15,16]. The current study demonstrated the presence of triterpenoids, including oleanolic acid, ursolic acid, the representative pentacyclic triterpenes, and their derivatives (**1**–**8**). The anthelmintic activity of *L. asiatica* is thought to be exerted by triterpenoids because several reports demonstrated that oleanolic acid and ursolic acid possessed anthelmintic activity [39,40], and saponins were also associated with the ovicidal action of the plant extract [41]. Thus, this study supports the evidence for clarifying the anthelmintic effect of *L. asiatica*. The antioxidant properties of the *L. asiatica* extract were well-proven in previous literatures and, especially the ethyl acetate-soluble extract, showed the most potent antioxidative effects [16]. In this study, eight flavonoids (**9**–**16**) were determined specifically from the ethyl acetate-soluble extract (Supplementary Information SI 9), which indicated that flavonoids were enriched in an ethyl acetate-soluble extract. Therefore, it is suggested that the antioxidant potency of the ethyl acetate-soluble extract of *L. asiatica* was directly related to the flavonoid contents because flavonoids such as catechins, quercetin, kaempferol, and myricetin and their derivatives are the most important natural antioxidants and exhibit numerous antioxidant-related beneficial effects [42,43,44,45,46]. Consequently, this study provides reasonable evidence for previous biological effects of *L. asiatica* as well as its phytochemical profile.

### 2.2. Characterisation of Compounds ***1***–***24***

*4-Hydroxyphenol-{6-O-[4-O-(7S,8R-guaiacylglycerol-8-yl)-3-methoxybenzoyl]}-β-D-glucopyra- noside* (**1**): Brown amorphous powder; ESI-Q-TOF-MS: *m/z* = 617.1884 [M − H]^−^ (calcd. for C_30_H_33_O_14_, 617.1870); [α]^22^_D_ -4.4 (*c* = 0.05, MeOH); CD(MeOH) *Δε* −5.35 (229 nm), −0.3 (252 nm), −0.14 (264 nm), −1.02 (281 nm), 0.05 (298 nm); UV (MeOH) λ_max_ (log ε) 204 (6.15), 219 (5.90), 266 (5.54), 285 (5.46) nm; ^1^H-NMR (500 MHz, MeOH-*d*_4_): δ 7.57 (1H, dd, *J* = 8.5, 2.0 Hz, H-6′′), 7.51 (1H, d, *J* = 2.0 Hz, H-2′′), 7.07 (1H, o, H-2′′′), 7.06 (1H, d, *J* = 8.5 Hz, H-5′′), 6.91 (2H, d, *J* = 9.0 Hz, H-2, 6), 6.87 (1H, dd, *J* = 8.1, 2.2 Hz, H-6′′′), 6.71 (1H, d, *J* = 8.1 Hz, H-5′′′), 6.56 (2H, d, *J* = 9.0 Hz, H-3, 5), 4.84 (1H, o, H-7′′′), 4.73 (1H, d, *J* = 7.4 Hz, H-1′), 4.68 (1H, dd, *J* = 11.7, 2.1 Hz, H-6′a), 4.64 (1H, m, H-8′′′), 4.37 (1H, dd, *J* = 11.7, 7.6 Hz, H-6′b), 3.86 (1H, brd, *J* = 4.9 Hz, H-9′′′), 3.80 (3H, s, 3′′′-OCH_3_), 3.79 (3H, s, 3″-OCH_3_), 3.71 (1H, m, H-5′), 3.45 (1H, o, H-3′), 3.44 (1H, o, H-2′), 3.39 (1H, brt, H-4′), m: Multiplet, o: Resonance was overlapped; ^13^C-NMR (125 MHz, MeOH-*d*_4_): δ 167.7 (C-7″), 154.2 (C-4″), 154.0 (C-4), 152.3 (C-1), 151.2 (C-3″), 148.8 (C-3″′), 147.2 (C-4″′), 133.9 (C-1″′), 124.8 (C-6″), 124.2 (C-1″), 121.4 (C-6″′), 119.6 (C-2), 119.6 (C-6), 116.7 (C-3), 116.7 (C-5), 116.4 (C-5″), 115.7 (C-5″′), 114.5 (C-2″), 112.2 (C-2″′), 103.7 (C-1′), 85.3 (C-8″′), 78.1 (C-3′), 75.6 (C-5′), 75.1 (C-2′), 74.3 (C-7″′), 72.2 (C-4′), 65.4 (C-6′), 62.6 (C-9″′), 56.7 (3″-OCH_3_), 56.5 (3″′-OCH_3_).

*Oleanolic acid* (**2**): White amorphous powder; ESI-Q-TOF-MS: *m/z* 455.3532 [M − H]^−^ (calcd. for C_30_H_47_O_3_, 455.3525); ^1^H-NMR (500 MHz, pyridine-*d*_5_): δ 5.52 (1H, s, H-12), 3.47 (1H, dd, *J* = 10.6, 6.4 Hz, H-3), 1.31 (3H, s, H-27), 1.27 (3H, s, H-23), 1.05 (6H, s, H-26, H-30), 1.03 (3H, s, H-24), 0.97 (3H, s, H-29), 0.92 (3H, s, H-25); ^13^C-NMR (125 MHz, pyridine-*d*_5_): δ 180.6 (C-28), 145.2 (C-13), 122.9 (C-12), 78.4 (C-3), 56.1 (C-5), 48.5 (C-9), 47.0 (C-17), 46.8 (C-19), 42.5 (C-14), 42.4 (C-18), 40.1 (C-8), 39.7 (C-4), 39.3 (C-1), 37.7 (C-10), 34.6 (C-21), 33.6 (C-22, 29), 33.5 (C-7), 31.3 (C-20), 29.1 (C-23), 28.6 (C-15), 28.4 (C-2), 26.5 (C-27), 24.2 (C-11), 24.1 (C-16), 23.2 (C-30), 19.1 (C-6), 17.8 (C-26), 16.9 (C-24), 15.9 (C-25).

Ursolic acid (**3**): White amorphous powder; ESI-Q-TOF-MS: *m/z* 455.3529 [M − H]^−^ (calcd. for C_30_H_47_O_3_, 455.3525); ^1^H-NMR (500 MHz, pyridine-*d*_5_): δ 5.51 (1H, s, H-12), 3.48 (1H, dd, *J* = 10.4, 5.7 Hz, H-3), 1.27 (6H, s, H-23, 26), 1.25 (3H, s, H-27), 1.05 (3H, s, H-24), 1.03 (3H, s, H-29), 0.98 (3H, s, H-30), 0.92 (3H, s, H-25); ^13^C-NMR (125 MHz, pyridine-*d*_5_): δ 180.4 (C-28), 139.6 (C-13), 125.9 (C-12), 78.4 (C-3), 56.1 (C-5), 53.9 (C-18), 48.4 (C-9), 48.4 (C-17), 42.8 (C-14), 40.3 (C-8), 39.7 (C-4, 19, 20), 39.4 (C-1), 37.7 (C-10), 37.6 (C-22), 33.9 (C-7), 31.4 (C-21), 29.1 (C-23), 29.0 (C-15), 28.4 (C-2), 25.3 (C-16), 24.2 (C-27), 23.9 (C-11), 21.7 (C-30), 19.1 (C-6), 17.8 (C-26), 16.9 (C-29), 16.0 (C-24), 14.6 (C-25).

*Maslinic acid* (**4**): White amorphous powder; ESI-Q-TOF-MS: *m/z* 471.3477 [M − H]^−^ (calcd. for C_30_H_47_O_4_, 471.3474); ^1^H-NMR (500 MHz, pyridine-*d*_5_): δ 5.49 (1H, s, H-12), 4.12 (1H, m, H-2), 3.42 (1H, d, *J* = 9.4 Hz, H-3), 1.30 (3H, s, H-29), 1.29 (3H, s, H-23), 1.23 (3H, s, H-27), 1.10 (3H, s, H-26), 1.04 (3H, s, H-30), 0.96 (3H, s, H-25); ^13^C-NMR (125 MHz, pyridine-*d*_5_): δ 180.7 (C-28), 145.3 (C-13), 122.7 (C-12), 84.1 (C-3), 68.9 (C-2), 56.2 (C-5), 48.5 (C-8), 48.5 (C-9), 48.1 (C-1), 47.0 (C-17), 46.8 (C-19), 42.5 (C-14), 42.3 (C-18), 40.2 (C-4), 38.9 (C-10), 34.6 (C-21), 33.6 (C-7), 33.5 (C-22, 29), 31.3 (C-20), 29.7 (C-23), 28.6 (C-15), 26.5 (C-27), 24.3 (C-30), 24.1 (C-11), 24.0 (C-16), 19.2 (C-6), 18.0 (C-26), 17.8 (C-25), 17.2 (C-24).

*Chebuloside ii* (**5**): White amorphous powder; ESI-Q-TOF-MS: *m/z* 689.3886 [M + Na]^+^ (calcd. for C_36_H_58_O_11_Na, 689.3877); ^1^H-NMR (500 MHz, pyridine-*d*_5_): δ 6.34 (1H, d, *J* = 8.1 Hz, H-1′), 5.55 (1H, t, *J* = 3.4 Hz, H-12), 5.12 (1H, s, H-6), 4.44 (2H, m, H-2, H-23), 4.26 (1H, m, H-3), 4.09 (1H, d, *J* = 10.6 Hz, H-23), 1.84 (3H, s, H-26), 1.79 (3H, s, H-23), 1.77 (3H, s, H-25), 1.22 (3H, s, H-27), 0.89 (6H, s, H-29, H-30); ^13^C-NMR (125 MHz, pyridine-*d*_5_): δ 176.7 (C-28), 143.8 (C-13), 123.4 (C-12), 96.2 (C-1′), 79.1 (C-3′), 78.7 (C-3, 5′), 74.5 (C-2′), 71.5 (C-4′), 69.4 (C-2), 67.9 (C-6), 66.6 (C-23), 62.5 (C-6′), 50.1 (C-1), 49.2 (C-9), 49.1 (C-5, 19), 47.3 (C-17), 43.2 (C-4, 14), 42.2 (C-18), 41.5 (C-7, 8), 38.6 (C-10), 34.4 (C-21), 33.4 (C-29), 32.9 (C-22), 31.1 (C-20), 28.6 (C-15), 26.5 (C-27), 23.9 (C-16, 30), 23.9 (C-30), 23.2 (C-11), 19.4 (C-26), 19.2 (C-25), 16.4 (C-24).

*Corosolic acid (***6**): White amorphous powder; ESI-Q-TOF-MS: *m/z* 471.3483 [M − H]^−^ (calcd. for C_30_H_47_O_4_, 471.3474); ^1^H-NMR (500 MHz, pyridine-*d*_5_): δ 5.48 (1H, s, H-12), 4.12 (1H, m, H-2), 3.42 (1H, d, *J* = 9.4 Hz, H-3), 1.30 (3H, s, H-29), 1.23 (3H, s, H-27), 1.10 (3H, s, H-24), 1.06 (3H, s, H-25), 1.00 (3H, d, *J* = 6.3 Hz, H-30), 0.97 (3H, d, *J* = 6.3 Hz, H-29); ^13^C-NMR (125 MHz, pyridine-*d*_5_): δ 180.4 (C-28), 139.7 (C-13), 125.8 (C-12), 84.1 (C-3), 68.9 (C-2), 56.2 (C-5), 53.9 (C-18), 48.4 (C-1, 17), 48.3 (C-9), 42.9 (C-14), 40.3 (C-8), 40.2 (C-4), 39.8 (C-20), 39.7 (C-19), 38.8 (C-10), 37.8 (C-22), 33.8 (C-7), 31.4 (C-21), 29.7 (C-23), 29.0 (C-15), 25.2 (C-16), 24.2 (C-27), 24.1 (C-11), 21.7 (C-30), 19.2 (C-6), 18.0 (C-24), 17.8 (C-26, 29), 17.3 (C-25).

*Hederagenin-3-O-arabinopyranoside* (**7**): White amorphous powder; ESI-Q-TOF-MS: *m/z* 663.3887 [M − H]^−^ (calcd. for C_35_H_55_O_8_, 603.3897); ^1^H-NMR (500 MHz, pyridine-*d*_5_): δ 5.49 (1H, s, H-12), 5.00 (1H, d, *J* = 7.1 Hz, H-1′), 4.31 (2H, m, H-3, H-23a), 3.75 (1H, d, *J* = 10.4 Hz, H-23b), 1.25 (3H, s, H-27), 1.04 (3H, s, H-26), 1.02 (3H, s, H-30), 0.96 (3H, s, H-25), 0.94 (6H, s, H-24, H-29); ^13^C-NMR (125 MHz, pyridine-*d*_5_): δ 180.7 (C-28), 145.2 (C-13), 122.8 (C-12), 107.0 (C-1′), 82.2 (C-3), 75.0 (C-2′), 73.4 (C-3′), 70.1 (C-4′), 67.3 (C-5′), 64.8 (C-23), 48.5 (C-9), 47.9 (C-5), 47.0 (C-17), 46.8 (C-19), 43.8 (C-4), 42.5 (C-14), 42.3 (C-18), 40.1 (C-8), 39.1 (C-1), 37.3 (C-10), 34.6 (C-21), 33.6 (C-22, 29), 33.2 (C-7), 31.3 (C-20), 28.7 (C-15), 26.5 (C-2, 27), 24.2 (C-11), 24.1 (C-16), 24.0 (C-30), 18.5 (C-6), 17.8 (C-26), 16.4 (C-25), 13.9 (C-24).

*Oleanolic acid 3-O-glucopyranosyl-(1→2)-arabinopyranoside* (**8**): White amorphous powder; ESI-Q-TOF-MS: *m/z* 749.4456 [M − H]^−^ (calcd. for C_41_H_65_O_12_, 749.4476); ^1^H-NMR (500 MHz, pyridine-*d*_5_): δ 5.49 (1H, s, H-12), 5.22 (1H, d, *J =* 7.7 Hz, H-1′′), 4.99 (1H, d, *J* = 5.9 Hz, H-1′), 3.22 (1H, dd, *J* = 11.6, 4.2 Hz, H-3), 1.30 (3H, s, H-27), 1.25 (3H, s, H-23), 1.06 (3H, s, H-24), 1.03 (6H, s, H-26, H-30), 0.97 (3H, s, H-29), 0.84 (3H, s, H-25); ^13^C-NMR (125 MHz, pyridine-*d*_5_): δ 180.6 (C-28), 145.2 (C-13), 122.8 (C-12), 106.4 (C-6′), 105.2 (C-1′), 89.1 (C-3), 81.4 (C-2′), 78.5 (C-2′′, 4′′), 76.8 (C-1′′), 73.8 (C-3′), 71.8 (C-3′′), 68.6 (C-4′), 65.3 (C-5′), 62.9 (C-5′′), 56.1 (C-5), 48.3 (C-9), 46.8 (C-17, 19), 42.5 (C-14), 42.3 (C-18), 40.0 (C-8), 39.8 (C-4), 39.0 (C-1), 37.3 (C-10), 34.5 (C-21), 33.6 (C-7, 22, 29), 31.3 (C-20), 28.6 (C-15, 23), 26.5 (C-2, 27), 24.1 (C-16), 24.0 (C-11, 30), 18.8 (C-6), 17.7 (C-26), 17.1 (C-24), 15.8 (C-25).

*(+)-Catechin* (**9**): Brown amorphous solid; ESI-Q-TOF-MS: *m/z* 291.0858 [M+H]^+^ (calcd. for C_15_H_15_O_6_, 291.0869); ^1^H-NMR (500 MHz, DMSO-*d*_6_): δ 6.84 (1H, s, H-2′), 6.76 (1H, d, *J* = 8.0 Hz, H-5′), 6.72 (1H, d, *J* = 8.0 Hz, H-6′), 5.93 (1H, d, *J* = 1.9 Hz, H-8), 5.85 (1H, d, *J* = 1.9 Hz, H-6), 4.56 (1H, d, *J* = 7.5 Hz, H-2), 3.97 (1H, dd, *J* = 13.2, 7.6 Hz, H-3), 2.85 (1H, dd, *J* = 16.1, 5.3 Hz, H-4a), 2.50 (1H, dd, *J* = 16.1, 8.1 Hz, H-4b); ^13^C-NMR (125 MHz, DMSO-*d*_6_): δ 156.4 (C-7), 156.1 (C-5), 155.3 (C-9), 144.8 (C-3′, 4′), 130.5 (C-1′), 118.4 (C-6′), 115.0 (C-5′), 114.5 (C-2′), 99.0 (C-10), 95.0 (C-6), 93.8 (C-8), 80.9 (C-2), 66.3 (C-3), 27.8 (C-4).

*(−)-Epicatechin* (**10**): Brown amorphous solid; ESI-Q-TOF-MS: *m/z* 291.0860 [M+H]^+^ (calcd. for C_15_H_15_O_6_, 291.0869); ^1^H-NMR (500 MHz, DMSO-*d*_6_): δ 6.88 (1H, d, *J* = 1.6 Hz, H-2′), 6.66 (1H, d, *J* = 8.1 Hz, H-5′), 6.64 (1H, dd, *J* = 8.1, 1.6 Hz, H-6′), 5.89 (1H, d, *J* = 2.3 Hz, H-6), 5.85 (1H, d, *J* = 2.3 Hz, H-8), 4.73 (1H, s, H-2), 4.00 (1H, s, H-3), 2.67 (1H, dd, *J* = 16.3, 4.5 Hz, H-4a), 2.49 (1H, dd, *J* = 16.3, 3.4 Hz, H-4b); ^13^C-NMR (125 MHz, DMSO-*d*_6_): δ 156.5 (C-5), 156.2 (C-7), 155.7 (C-9), 144.4 (C-3′, 4′), 130.5 (C-1′), 117.9 (C-6′), 114.8 (C-5′), 114.7 (C-2′), 98.4 (C-10), 95.0 (C-6), 94.0 (C-8), 78.0 (C-2), 64.8 (C-3), 28.1 (C-4).

*(−)-Epiafzelechin* (**11**): Yellow amorphous powder; ESI-Q-TOF-MS: *m/z* 297.0735 [M + Na]^+^ (calcd. for C_15_H_14_O_5_Na, 297.0739); ^1^H-NMR (500 MHz, DMSO-*d*_6_): δ 7.22 (2H, d, *J* = 8.5 Hz, H-2′, 6′), 6.71 (2H, d, *J* = 8.5 Hz, H-3′, 5′), 5.89 (1H, d, *J* = 2.3 Hz, H-8), 5.72 (1H, d, *J* = 2.3 Hz, H-6), 4.80 (1H, s, H-2), 4.02 (1H, dd, *J* = 8.3, 4.5 Hz, H-3), 2.67 (1H, m, H-4a), 2.46 (1H, m, H-4b); ^13^C-NMR (125 MHz, DMSO-*d*_6_): δ 156.5 (C-7), 156.2 (C-5, 9), 155.7 (C-4′), 129.9 (C-1′), 128.2 (C-2′, 6′), 114.3 (C-3′, 5′), 98.4 (C-10), 95.0 (C-8), 94.0 (C-6), 77.9 (C-2), 64.7 (C-3), 28.1 (C-4).

*Juglanin* (**12**): Yellow amorphous powder; ESI-Q-TOF-MS: *m/z* 441.0796 [M + Na]^+^ (calcd. for C_20_H_18_O_10_Na, 441.0798); ^1^H-NMR (500 MHz, DMSO-*d*_6_): δ 7.96 (2H, d, *J* = 8.7 Hz, H-2′, 6′), 6.92 (2H, d, *J* = 8.7 Hz, H-3′, 5′), 6.40 (1H, d, *J* = 1.6 Hz, H-8), 6.21 (1H, d, *J* = 1.6 Hz, H-6), 5.50 (1H, s, H-1′′); ^13^C-NMR (125 MHz, DMSO-*d_6_*): δ 180.0 (C-4), 166.2 (C-7), 163.2 (C-5), 161.7 (C-4′), 159.5 (C-9), 158.7 (C-2), 135.1 (C-3), 132.1 (C-2′, 6′), 122.9 (C-1′), 116.6 (C-3′, 5′), 109.8 (C-1′′), 105.8 (C-10), 100.0 (C-6), 94.9 (C-8), 88.1 C-4′′), 83.5 (C-2′′), 78.8 (C-3′′), 62.7 (C-5′′).

*Mearnsetin 3-O-rhamnopyranoside* (**13**): Yellow amorphous powder; ESI-Q-TOF-MS: *m/z* 501.1012 [M + Na]^+^ (calcd. for C_22_H_22_O_12_, 501.1009); ^1^H-NMR (500 MHz, DMSO-*d*_6_): δ 6.81 (2H, s, H-2′, 6′), 6.36 (1H, brs, H-6), 6.19 (1H, brs, H-8), 5.15 (1H, brs, H-1′′), 3.98 (1H, dd, 3.5, 1.2 Hz, H-2′′), 3.74 (1H, s, 4′-OCH_3_), 3.51 (1H, dd, J = 8.4, 3.0 Hz, H-3′′), 3.32 (1H, m, H-5′′), 3.15 (1H, m, H-4′′), 0.82 (3H, d, *J* = 5.4 Hz, H-6′′); ^13^C-NMR (125 MHz, DMSO-*d*_6_): δ 177.7 (C-4), 164.9 (C-7), 161.3 (C-5), 157.1 (C-9), 156.5 (C-2), 150.7 (C-3′, 5′), 137.7 (C-4′), 134.8 (C-3), 124.8 (C-1′), 108.1 (C-2′, 6′), 103.9 (C-10), 102.1 (C-1′′), 98.9 (C-6), 93.7 (C-8), 71.1 (C-4′′), 70.5 (C-3′′), 70.3 (C-2′′), 70.0 (C-5′′), 59.7 (4′-OCH_3_), 17.4 (C-6′′).

*Myricitrin* (**14**): Yellow amorphous powder; ESI-Q-TOF-MS: *m/z* 487.0852 [M + Na]^+^ (calcd. for C_21_H_20_O_12_, 487.0852); ^1^H-NMR (500 MHz, DMSO-*d*_6_): δ 6.90 (2H, s, H-2′, 6′), 6.38 (1H, d, *J* = 1.9 Hz, H-8), 6.21 (1H, d, *J* = 1.9 Hz, H-6), 5.21 (1H, d, *J* = 1.0 Hz, H-1′′), 3.99 (1H, brs, H-2′′), 3.57 (1H, dd, *J* = 9.2, 3.0 Hz, H-3′′), 3.40 (1H, m, H-5′′), 3.17 (1H, m, H-4′′) 0.86 (3H, d, *J* = 6.2 Hz, H-6′′); ^13^C-NMR (125 MHz, DMSO-*d*_6_): δ 177.7 (C-4), 164.2 (C-7), 161.2 (C-5), 157.4 (C-2), 156. 3(C-9), 145.7 (C-3′, 5′), 136.4 (C-4′), 134.2 (C-3), 119.5 (C-1′), 107.8 (C-2′, 6′), 103.9 (C-10), 101.9 (C-1′′), 98.6 (C-6), 93.4 (C-8), 71.2 (C-4′′), 70.5 (C-5′′), 70.3 (C-3′′), 69.9 (C-2′′), 17.5 (C-6′′).

*Afzelin* (**15**): Yellow amorphous powder; ESI-Q-TOF-MS: *m/z* 455.0954 [M + Na]^+^ (calcd. for C_21_H_20_O_10_, 455.0954); ^1^H-NMR (500 MHz, DMSO-*d*_6_): δ 7.75 (2H, d, *J* = 8.7 Hz, H-2′, 6′), 6.90 (2H, d, *J* = 8.7 Hz, H-3′, 5′), 6.33 (1H, s, H-8), 6.14 (1H, s, H-6), 5.29 (1H, s, H-1′′), 3.97 (1H, brs, H-2′′), 3.47 (1H, dd, *J* = 9.0, 2.9 Hz, H-3′′), 3.38 (1H, o, H-5′′), 3.13 (1H, t, *J* = 9.2 Hz, H-4′′), 0.79 (3H, d, *J* = 6.0 Hz, H-6′′); ^13^C-NMR (125 MHz, DMSO-*d*_6_): δ 177.4 (C-4), 164.7 (C-7), 161.2 (C-5), 159.9 (C-4′), 158.8 (C-9), 156.6 (C-2), 134.0 (C-3), 130.5 (C-2′, 6′), 120.5 (C-1′), 115.3 (C-3′, 5′), 104.3 (C-10), 101.7 (C-1′′), 99.0 (C-6), 93.9 (C-8), 70.5 (C-3′′), 70.3 (C-2′′), 70.1 (C-4′′), 70.0 (C-5′′), 17.4 (C-6′′).

*Quercitrin* (**16**): Yellow amorphous powder; ESI-Q-TOF-MS: *m/z* 447.0931 [M − H]^−^ (calcd. for C_21_H_19_O_11_, 447.0927); ^1^H-NMR (500 MHz, DMSO-*d*_6_): δ 7.30 (1H, d, *J* = 2.1 Hz, H-2′), 7.26 (1H, dd, *J* = 8.3, 2.1 Hz, H-6′), 6.87 (1H, d, *J* = 8.3 Hz, H-5′), 6.38 (1H, s, H-6), 6.20 (1H, s, H-8), 5.27 (1H, s, H-1′′), 3.99 (1H, brs, H-2′′), 3.52 (1H, dd, *J* = 9.1, 3.3 Hz, H-3′′), 3.40 (1H, o, H-5′′), 3.16 (1H, t, *J* = 9.4 Hz, H-4′′), 0.83 (3H, d, *J* = 6.1 Hz, H-6′′); ^13^C-NMR (125 MHz, DMSO-*d*_6_): δ 177.6 (C-4), 164.7 (C-7), 161.2 (C-5), 157.1 (C-9), 156.4 (C-2), 148.5 (C-4′), 145.2 (C-3′), 134.1 (C-3), 121.0 (C-6′), 120.6 (C-1′), 115.5 (C-5′), 115.4 (C-2′), 103.9 (C-10), 101.7 (C-1′′), 98.8 (C-6), 93.6 (C-8), 71.1 (C-4′′), 70.5 (C-3′′), 70.3 (C-2′′), 70.0 (C-5′′), 17.4 (C-6′′).

*4-Hydroxyphenol-[6-O-(4′′-hydroxy-3′′,5′′-dimethoxy-benzoate)]-β-D-glucopyranoside* (**17**): Brown amorphous solid; ESI-Q-TOF-MS *m/z* 475.1217 [M+H]^+^ (calcd. for C_21_H_25_O_11_, 475.1216); ^1^H-NMR (500 MHz, MeOH-*d*_4_): δ 7.35 (2H, s, H-2′′, 6′′), 6.91 (2H, d, *J* = 8.9 Hz, H-2, 6), 6.58 (2H, d, *J* = 8.9 Hz, H-3, 5), 6.69 (1H, *J* = 12.2 Hz, H-6′a), 4.74 (1H, d, *J* = 7.4 Hz, H-1′), 4.38 (1H, d, *J* = 12.1 Hz, H-6′b), 3.86 (6H, s, 3′′-OCH_3_ and 5′′-OCH_3_), 3.39-3.72 (4H, m, H-2′-5′),; ^13^C-NMR (125 MHz, MeOH-*d*_4_): δ 168.0 (C-7′′), 154.0 (C-4), 152.4 (C-1), 149.1 (C-3′′, 5′′), 142.5 (C-4′′), 125.4 (C-1′′), 119.6 (C-2, 6), 116.7 (C-3, 5), 108.5 (C-2′′, 6′′), 103.8 (C-1′), 78.0 (C-3′), 75.7 (C-5′), 75.1 (C-2′), 72.2 (C-4′), 65.4 (C-6′), 57.1 (3′′-OCH_3_ and 5′′-OCH_3_)

*Breynioside A* (**18**): Brown amorphous solid; ESI-Q-TOF-MS *m/z* 391.1043 [M − H]^−^ (calcd. for C_19_H_20_O_9_, 391.1029); ^1^H-NMR (500 MHz, MeOH-*d*_4_): δ 7.90 (2H, d, *J* = 8.7 Hz, H-2′, 6′), 6.94 (2H, d, *J* = 8.9 Hz, H-2, 6), 6.86 (2H, d, *J* = 8.7 Hz, H-3′, 5′), 6.60 (2H, d, *J* = 8.9 Hz, H-3, 5), 3.41-3.87 (6H, m, H-2′-6′); ^13^C-NMR (125 MHz, MeOH-*d*_4_): δ 168.0 (C-7′′), 163.8 (C-4′′), 154.0 (C-4), 152.4 (C-1), 133.0 (C-6′′), 133.0 (C-2′′), 122.3 (C-1′′), 119.7 (C-6), 119.7 (C-2), 116.7 (C-3, 5), 116.4 (C-5′′), 116.4 (C-3′′), 103.8 (C-1′), 78.1 (C-3′), 75.7 (C-5′), 75.1 (C-2′), 72.2 (C-4′), 65.2 (C-6′).

*Phenylethyl-rutinoside* (**19**): White amorphous powder; ESI-Q-TOF-MS: *m/z* 453.1748 [M + Na]^+^ (calcd. for C_20_H_30_O_10_Na, 453.1737); ^1^H-NMR (500 MHz, MeOH-*d*_4_): δ 7.27 (2H, m, H-2, 6), 7.26 (2H, o, H-3, 5), 7.17 (1H, o, H-4), 4.74 (1H, d, *J* = 1.3 Hz, H-1′′), 4.29 (1H, d, *J* = 7.8 Hz, H-1′), 4.04 (1H, m, H-8a), 3.77 (1H, m, H-8b), 2.94 (2H, td, *J* = 7.3, 3.0 Hz, H-7), 1.25 (1H, d, *J* = 6.2 Hz, H-6′′); ^13^C-NMR (125 MHz, MeOH-*d*_4_): δ 140.1 (C-1), 130.1 (C-3, 5), 129.5 (C-2, 6), 127.3 (C-4), 104.7 (C-1′), 102.4 (C-1′′), 78.3 (C-3′), 77.1 (C-5′), 75.3 (C-2′), 74.2 (C-4′′), 72.5 (C-3′′), 72.3 (C-2′′), 71.9 (C-8), 71.8 (C-4′), 69.9 (C-5′′), 68.4 (C-6′), 37.4 (C-7), 18.5 (C-6′′).

*Icariside D1* (**20**): White powder; ESI-Q-TOF-MS: *m/z* 439.1581 [M + Na]^+^ (calcd. for C_19_H_28_O_10_Na, 439.1580); ^1^H-NMR (500 MHz, pyridine-*d_5_*): δ 7.29 (2H, o, H-2, 6), 7.27 (2H, o, H-3, 5), 7.17 (1H, o, H-4), 5.82 (1H, d, *J* = 2.5 Hz, H-1′′), 4.85 (1H, d, *J* = 7.8 Hz, H-1′), 4.35 (1H, m, H-8a), 3.91 (1H, m, H-8b), 3.00 (2H, t, *J* = 7.3 Hz, H-7); ^13^C-NMR (125 MHz, pyridine-*d_5_*): δ 139.7 (C-1), 129.8 (C-3, 5), 129.8 (C-5), 129.0 (C-2, 6), 126.7 (C-4), 111.5 (C-1′′), 105.0 (C-1′), 80.8 (C-3′′), 78.9 (C-3′), 78.1 (C-2′′), 77.5 (C-5′), 75.4 (C-2′), 75.4 (C-4′′), 70.9 (C-8, 4′), 69.3 (C-6′), 65.9 (C-5′′), 37.0 (C-7).

*Hexenyl-rutinoside* (**21**): White amorphous powder; ESI-Q-TOF-MS: *m/z* 431.1892 [M + Na]^+^ (calcd. for C_18_H_32_O_10_Na, 431.1893); ^1^H-NMR (500 MHz, MeOH-*d*_4_): δ 5.46 (1H, o, H-4), 5.40 (1H, o, H-3), 4.74 (1H, d, *J* = 1.3 Hz, H-1′′), 4.25 (1H, d, *J* = 7.8 Hz, H-1′), 3.81 (1H, m, H-1a), 3.54 (1H, m, H-1b), 2.38 (2H, m, H-2), 2.08 (2H, m, H-5), 1.26 (3H, d, *J* = 6.3 Hz, H-6′′); ^13^C-NMR (125 MHz, MeOH-*d*_4_): δ 134.7 (C-4), 126.1 (C-3), 104.6 (C-1′), 102.4 (C-1′′), 78.2 (C-3′), 77.0 (C-5′), 75.2 (C-2′), 74.2 (C-4′′), 72.5 (C-3′′), 72.4 (C-2′′), 71.8 (C-4′), 70.7 (C-1), 69.9 (C-5′′), 68.3 (C-6′), 28.9 (C-2), 21.6 (C-5), 18.1 (C-6′′), 14.7 (C-6).

*Everlastoside C* (**22**): Black powder; ESI-Q-TOF-MS: *m/z* 405.1741 [M + Na]^+^ (calcd. for C_16_H_30_O_10_Na, 405.1737); ^1^H-NMR (500 MHz, pyridine-*d*_5_): δ 5.83 (1H, d, *J* = 2.3 Hz, H-1′′), 4.81 (1H, d, *J* = 7.8 Hz, H-1′), 4.19 (1H, m, H-1a), 3.68 (1H, m, H-1b), 1.72 (1H, m, H-3), 1.52 (2H, dd, *J* = 13.4, 6.7 Hz, H-2), 0.82 (3H, d, *J* = 5.4 Hz, H-4), 0.80 (3H, d, *J* = 5.4 Hz, H-5); ^13^C-NMR (125 MHz, pyridine-*d*_5_): δ 111.5 (C-1′′), 105.0 (C-1′), 80.8 (C-3′′), 78.9 (C-3′), 78.1 (C-2′′), 77.5 (C-5′), 75.4 (C-2′), 75.4 (C-4′′), 70.9 (C-4′), 69.3 (C-6′), 68.5 (C-1), 65.9 (C-5′′), 39.3 (C-2), 25.4 (C-3), 23.0 (C-5), 22.9 (C-4).

*Berganin* (**23**): Brown powder; ESI-Q-TOF-MS: *m/z* 351.0701 [M + Na]^+^ (calcd. for C_14_H_16_O_9_Na, 351.0692); ^1^H-NMR (500 MHz, DMSO-*d*_6_): δ 7.01 (1H, s, H-7), 4.98 (1H, d, *J* = 10.8 Hz, H-10b), 3.99 (1H, dd, *J* = 10.8, 9.6 Hz, H-4a), 3.85 (1H, brd, *J* = 11.4 Hz, H-11a), 3.78 (1H, s, OMe), 3.64 (1H, brd, *J* = 10.3 Hz, H-4), 3.58 (1H, m, H-2), 3.44 (1H, o, H-11b), 3.19 (1H, t, *J* = 8.7 Hz, H-3); ^13^C-NMR (125 MHz, DMSO-*d*_6_): δ 163.8 (C-6), 151.4 (C-8), 148.5 (C-10), 141.0 (C-9), 118.5 (C-6a), 116.3 (C-10a), 109.9 (C-7), 82.2 (C-2), 80.2 (C-4a), 74.1 (C-4), 72.5 (C-10b), 71.1 (C-3), 61.5 (C-11).

*Citroside A* (**24**): Brown amorphous powder; ESI-Q-TOF-MS: *m/z* 409.1842 [M + Na]^+^ (calcd. for C_19_H_30_O_8_Na, 409.1838); ^1^H-NMR (500 MHz, MeOH-*d*_4_): δ 5.89 (1H, s, H-8), 4.52 (1H, d, *J* = 7.7 Hz, H-1′), 4.32 (1H, m, H-3), 2.48 (1H, ddd, *J* = 13.4, 4.0, 2.1 Hz, H-4a), 2.19 (3H, s, H-11), 1.92 (1H, d, *J* = 1.4 Hz, H-2a), 1.47 (3H, s, H-10), 1.38 (3H, s, H-12), 1.36 (3H, d, *J* = 2.3 Hz, H-4b), 1.34 (3H, d, *J* = 1.5 Hz, H-2b), 1.15 (3H, s, H-13); ^13^C-NMR (125 MHz, MeOH-*d*_4_): δ 213.1 (C-9), 200.8 (C-7), 119. 2(C-6), 101.5 (C-8), 98.8 (C-1′), 78.8 (C-5), 78.7 (C-3′), 77.9 (C-5′), 75.4 (C-2′), 71.8 (C-4′), 63.9 (C-3), 63.0 (C-6′), 50.0 (C-2), 48.2 (C-4), 37.1 (C-1), 32.6 (C-13), 30.2 (C-12), 26.8 (C-11), 26.7 (C-10).

## 3. Materials and Methods

### 3.1. General Experiments

^1^H-NMR (500 MHz) and ^13^C-NMR (125MHz) data were obtained using a Bruker Avance III 500 Spectrometer (Bruker, Karlsruhe, Germany). ESI-Q-TOF-MS spectra were acquired using an Agilent 6530 Accurate-Mass Q-TOF LC/MS system (Agilent Technologies, Santa Clara, CA, USA). Preparative-scale high performance liquid chromatography (HPLC) was performed using a Gilson HPLC system (Middleton, WI, USA) composed of a binary pump, a liquid handler, and a UV/VIS detector. Gas chromatography (GC) was performed using a GC353B-FSL (GL Sciences Inc., Tokyo, Japan) and a flame ionization detector. A Jasco P-2000 polarimeter and a J-815 CD spectrometer (Jasco, Tokyo, Japan) were used to record optical rotation and circular dichroism (CD) spectra, respectively. UV absorbance was recorded using a UV-1800 spectrophotometer (Shimadzu, Kyoto, Japan). Silica gel (40-63 μm, Merck, Germany), ODS resin (S-5 μm, YMC Co., Tokyo, Japan), and SephadexTM LH-20 (GE Healthcare, Chicago, IL, USA) were used in liquid column chromatography, and thin layer chromatography was performed using Silicagel 60 F254 (Merck, Darmstadt, Germany). The preparative HPLC column was a Luna C18 (21.2 × 250 mm I.D., 5 μm; Phenomenex, Torrance, CA, USA). A BPX50 column (0.25 mm × 30 m; Trajan Scientific and Medical, Victoria, Australia) was used to perform GC analysis. Authentic D-glucose was purchased from Sigma-Aldrich Korea (Yongin, Korea). Organic solvents used in extraction, partition, and column chromatography were of analytical grade and were purchased from Dae-Jung Chemical Co. Ltd. (Seoul, Korea). Methanol and acetonitrile for HPLC were provided by Thermo Fisher Scientific Korea (Seoul, Korea).

### 3.2. Plant Material

The aerial parts of *Leea asiatica* were collected from Popa Mountain National Park (Mandalay, Myanmar) in August 2011. Khin Myo Htwe (Popa Mountain National Park) identified specimens of *L. asiatica* and a voucher specimen (#PopaLeeaA082011) was deposited at the Herbarium of the College of Pharmacy, the Catholic University of Korea.

### 3.3. Extraction and Isolation

The dried *L. asiatica* (651.4 g) samples were treated with methanol and extracts were obtained through immersion in an ultrasonic bath (3 h × 3 times). This process yielded methanol-soluble extracts (35.3 g). The methanol-soluble extracts were suspended in water and partitioned sequentially with organic solvents to yield *n*-hexane- (11.2 g), ethyl acetate- (9.1 g), and *n*-butanol-soluble extracts (6.1 g). The detailed isolation schemes for compounds **1**–**24** from organic solvent soluble extracts are described in Supplementary Information SI 9.

### 3.4. Acid Hydrolysis of Compound ***1***

Acid hydrolysis of compound **1** was carried out based on the previous report [47]. Derivatized authentic D-glucose gave GC peaks at *t*_R_ 11.84 min and the *t*_R_ of hydrolysate of 1 was similar to that of authentic D-glucose.

## Supplementary Materials

1-, 2-dimentional NMR, ESI-Q-TOF-MS, CD, and UV spectroscopic data of **1** are available online.
