# Supplementary material for: Phytochemical Study of Aerial Parts of Leea asiatica
Source: Molecules. 2019 May 4;24(9):1733. doi: 10.3390/molecules24091733 (PMC6539499; doi:10.3390/molecules24091733)
Supplement: Supplementary file 1 [file molecules-24-01733-s001.pdf]

Supplementary Information

Phytochemical study of aerial parts of *Leea asiatica*

Hyun Woo Kil[a1,†], Taewoong Rho[1,†] and Kee Dong Yoon[1,*]

[1] College of Pharmacy and Integrated Research Institute of Pharmaceutical Sciences, The Catholic University of Korea, Bucheon 14662, Republic of Korea; karlwho@naver.com (T.R.); kilhyunwoo@catholic.ac.kr (H.W.K.); kdyoon@catholic.ac.kr (K.D.Y.)

* Correspondence: e-mail kdyoon@catholic.ac.kr; Tel.: +82-2-2164-4091

† These authors contributed equally to this work

Contents

SI 1: ^{1}H-NMR spectrum of compound **1**.

SI 2: ^{13}C-NMR spectrum of compound **1**.

SI 3: ^{1}H-^{1}H COSY spectrum of compound **1**.

SI 4: HSQC spectrum of compound **1**.

SI 5: HMBC spectrum of compound **1**.

SI 6: ESI-Q-TOF-MS spectrum of compound **1**.

SI 7: UV spectrum of compound **1**.

SI 8: Comparison of ^{1}H and ^{13}C NMR data between compound **1** and (-)-4-hydroxy-3-methoxyphenol β-D-{6-*O*-[4-*O*-(7*S*,8*R*)-(4-hydroxy-3-methoxyphenylglycerol-8-yl)-3-methoxybenzoyl]}-glucopyranoside [Ref. 17].

SI 9: Isolation scheme of compounds **1**–**24** from the aerial parts of *Leea asiatica*.

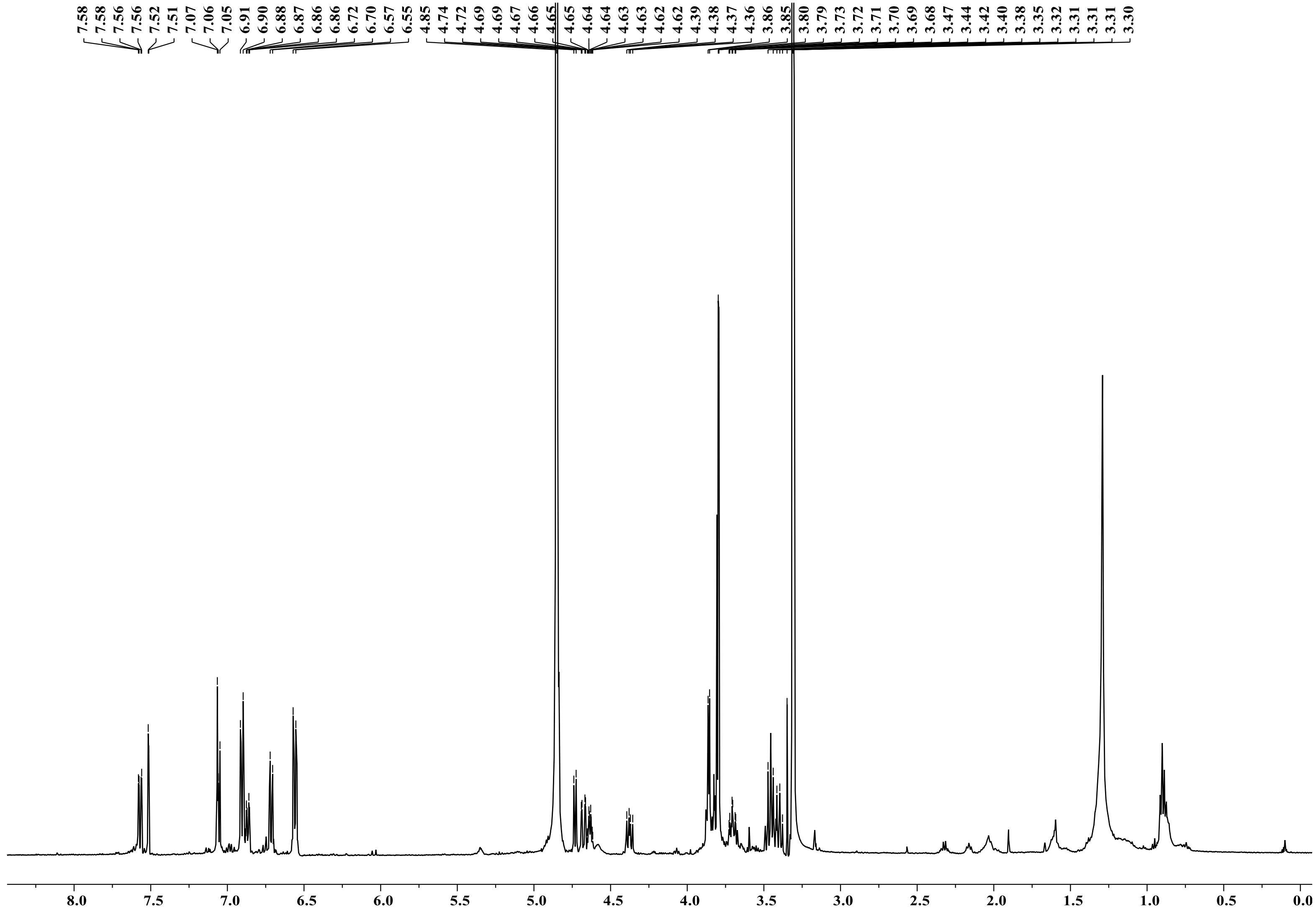

SI 1. ^{1}H-NMR spectrum of compound **1**.

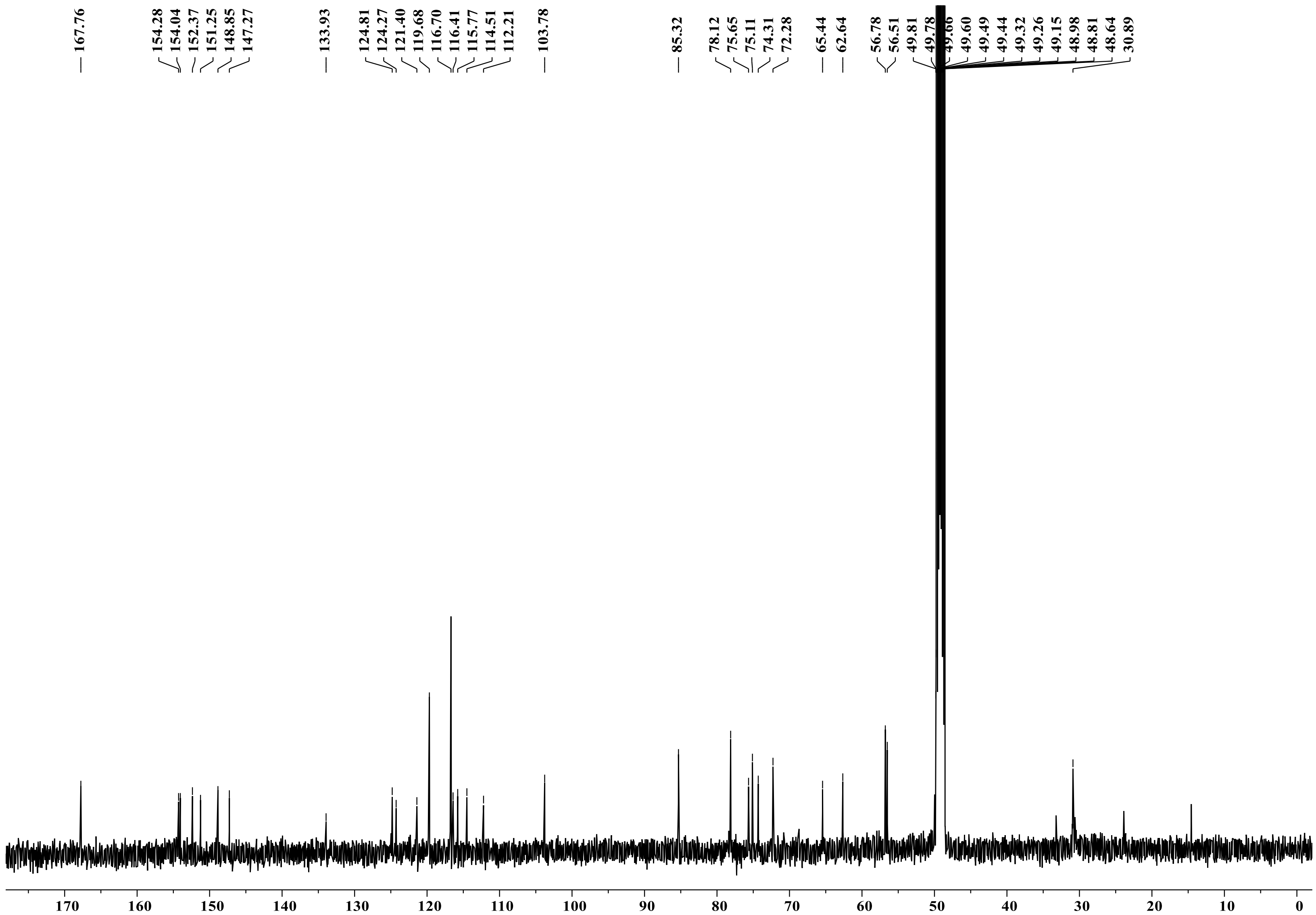

SI 2. ^{13}C-NMR spectrum of compound **1**.

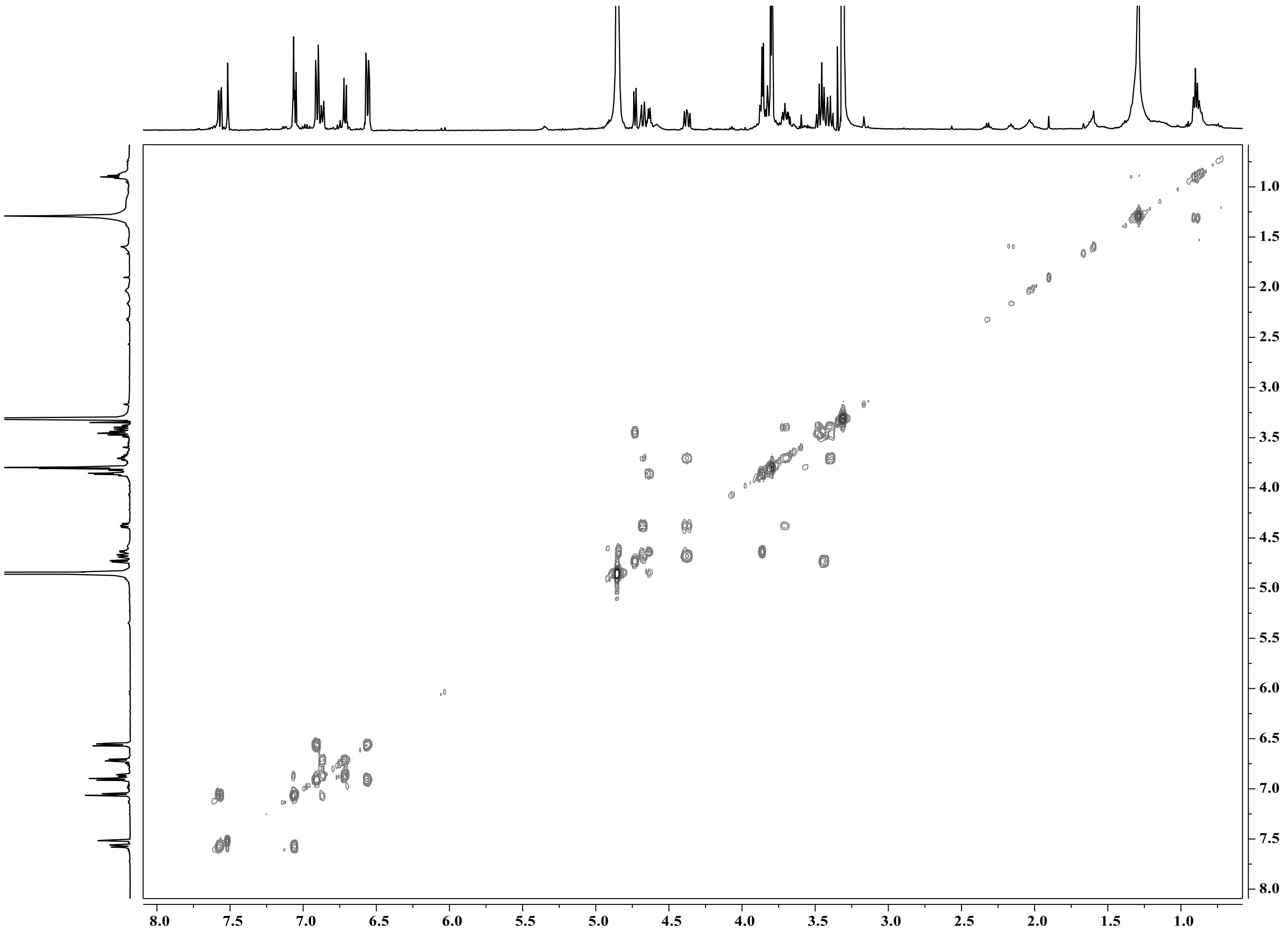

SI 3. ^{1}H-^{1}H COSY spectrum of compound **1**.

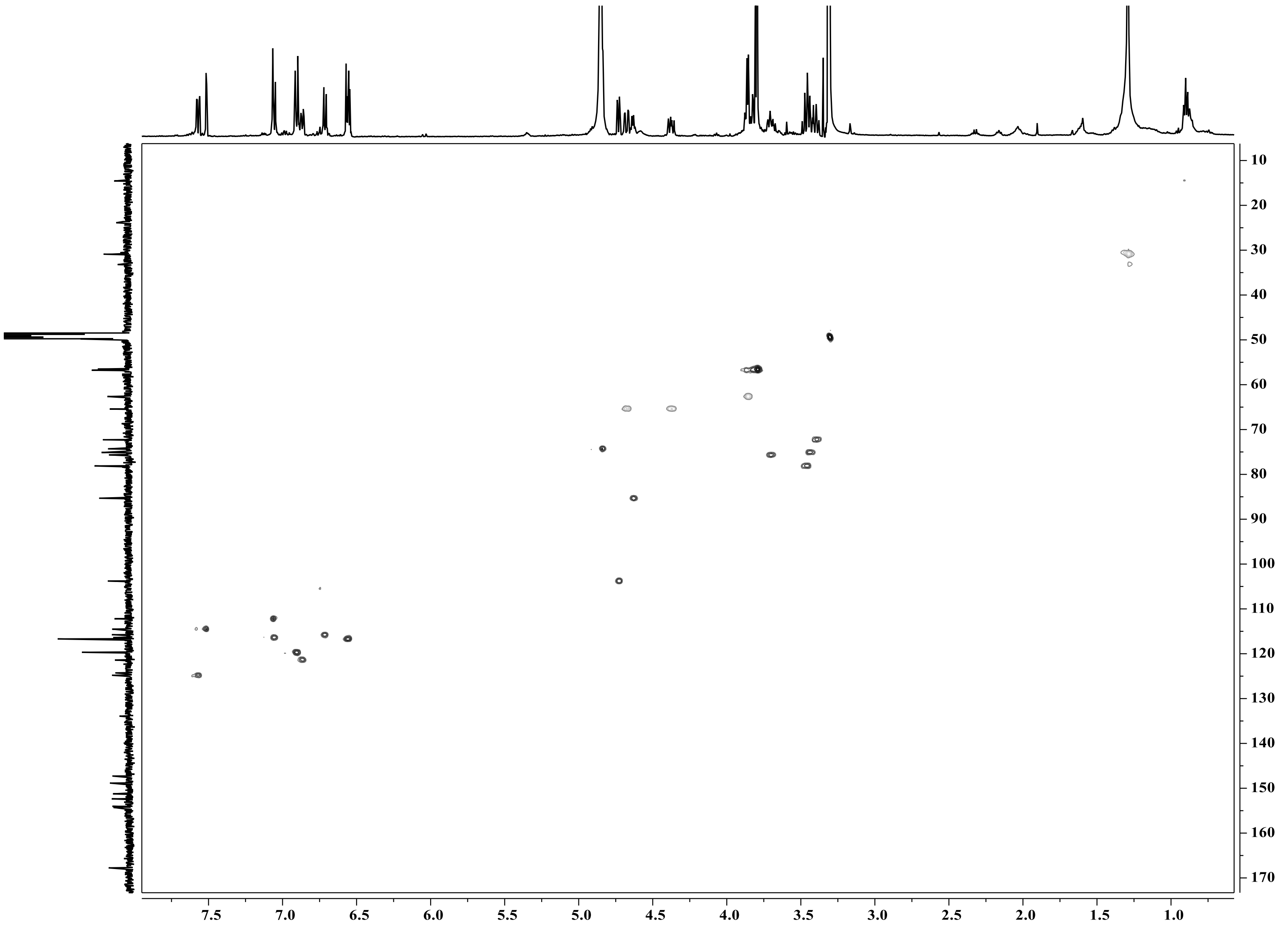

SI 4. HSQC spectrum of compound **1**.

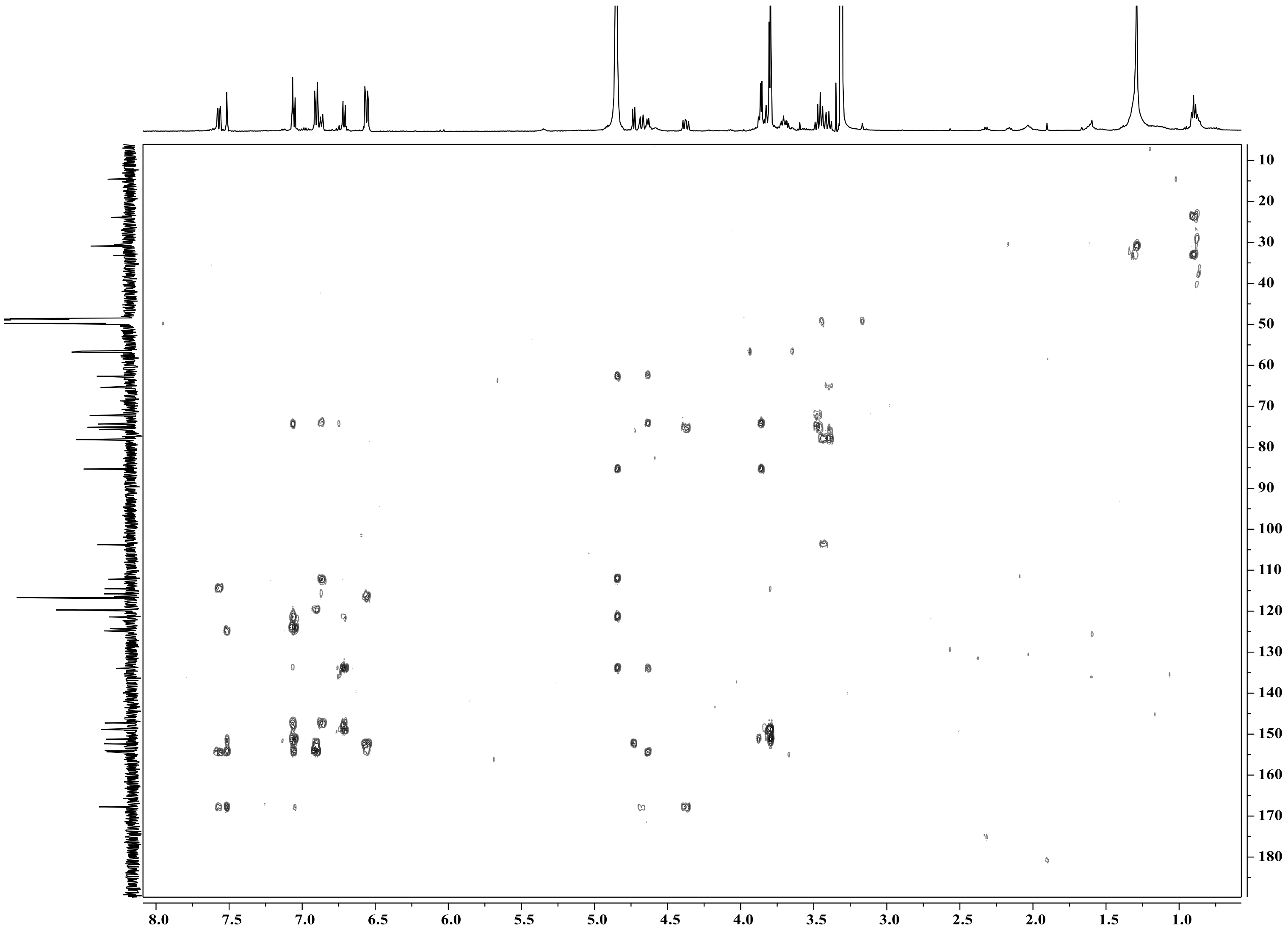

SI 5. HMBC spectrum of compound **1**.

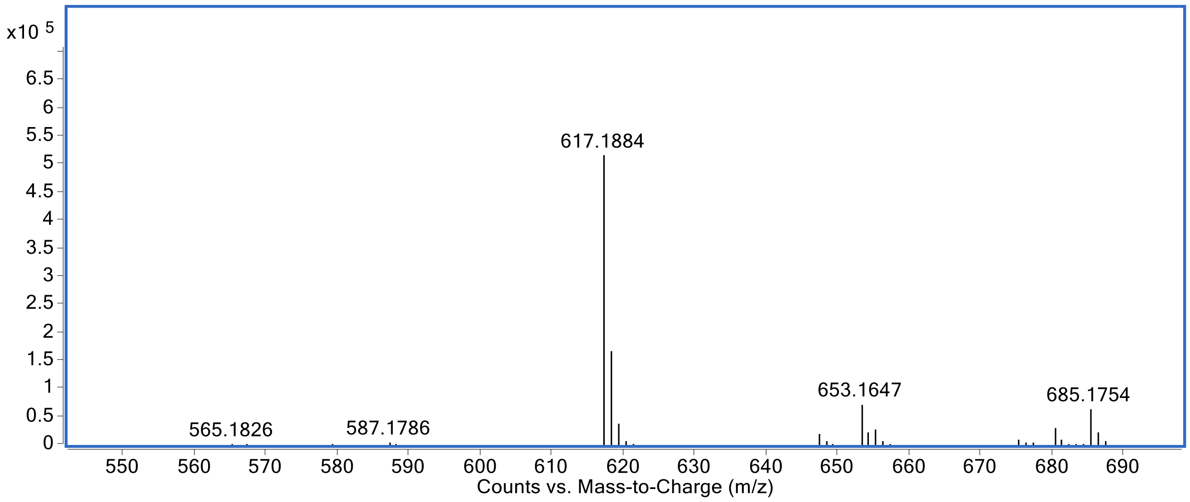

SI 6. ESI-Q-TOF-MS spectrum of compound **1**.

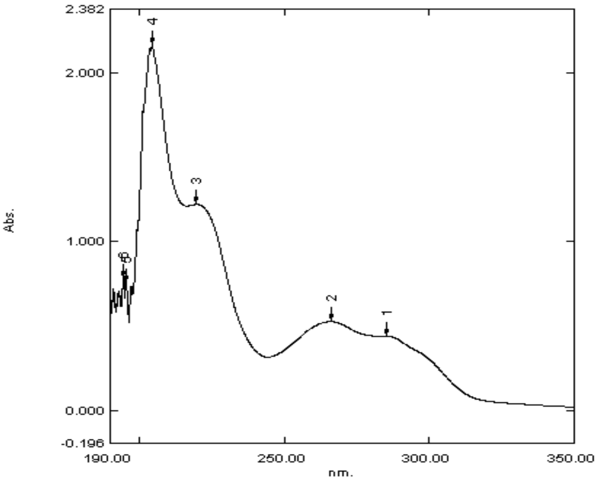

1: 285.10 / 0.437
2: 266.10 / 0.526
3: 219.80 / 1.222
4: 204.50 / 2.167

SI 7. UV spectrum of compound **1**.

SI 8. Comparison of ^{1}H- and ^{13}C-NMR data between compound **1** and (-)-4-hydroxy-3-methoxyphenol β-D-{6-*O*-[4-*O*-(7*S*,8*R*)-(4-hydroxy-3-methoxyphenylglycerol-8-yl)-3-methoxybenzoyl]}-glucopyranoside [Ref. 17]

**Compound 1***			**Reference compound (Ref. 16)***	
Position	^{1}H-NMR	^{13}C-NMR	^{1}H-NMR	^{13}C-NMR
1		152.3		152.5
2	6.91 d (9.0)	119.6	6.63 s	104.1
3	6.56 d (9.0)	116.7		149.2
4		154		143.1
5	6.56 d (9.0)	116.7	6.47 d (8.0)	115.9
6	6.91 d (9.0)	119.6	6.48 d (8.0)	110.2
1′	4.73 d (7.4)	103.7	4.70 d (7.5)	103.7
2′	3.44 o***	75.1	3.38 dd (8.5, 7.5)	75.0
3′	3.45 o***	78.1	3.42 t (8.5)	78.0
4′	3.39 brt (9.1)	72.2	3.34 t (8.5)	72.1
5′	3.71 m**	75.6	3.66 m**	75.6
6′	4.68 dd (11.7, 2.1), 4.37 dd (11.7, 7.6)	65.4	4.64 d (11.0), 4.34 dd (11.0, 6.0)	65.3
1″		124.2		124.2
2″	7.51 d (2.0)	114.5	7.49 s	114.2
3″		151.2		151.0
4″		154.2		154.4
5″	7.06 d (8.5)	116.4	7.04 d (8.5)	116.1
6″	7.57 dd (8.5, 2.0)	124.8	7.50 d (8.5)	124.8
7″		167.7		167.6
1‴		133.9		133.7
2‴	7.07 o***	112.2	7.00 s	111.7
3‴		148.8		148.9
4‴		147.2		147.2
5‴	6.71 d (8.1)	115.7	6.70 d (8.0)	115.9
6‴	6.87 dd (8.1, 2.2)	121.4	6.82 brd (8.0)	120.7
7‴	4.84 o***	74.3	4.85 d (5.0)	74.0
8‴	4.64 m**	85.3	4.52 m**	85.8
9‴	3.86 brd (4.9)	62.6	3.73 dd (12.0, 4.0), 3.50 dd (12.0, 6.0)	62.6
3-OMe			3.61 s	56.3
3″-OMe	3.79 s	56.7	3.81 s	56.6
3‴-OMe	3.80 s	56.5	3.77 s	56.4

*Compounds were dissolved in CD_3OD.

**m: multiplet

***o: resonance was overlapped.

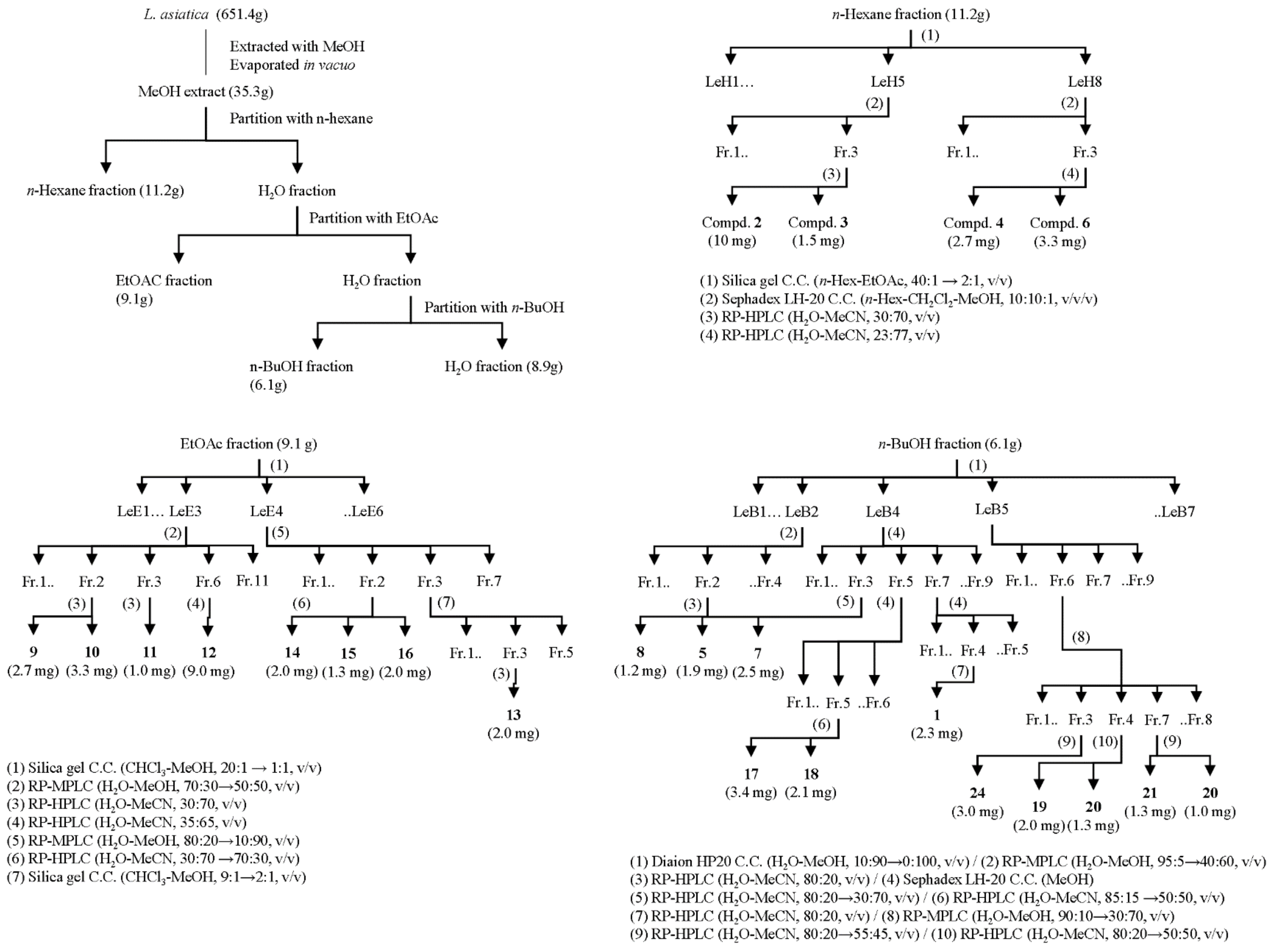

SI 9. Isolation scheme of compounds **1–24** from the aerial parts of *Leea asiatica*.
